# The epidemiology of intransient TB-induced hyperglycaemia in previously undiagnosed diabetes mellitus 2 individuals: a protocol for a systematic review and meta-analysis

**DOI:** 10.1186/s13643-019-1143-0

**Published:** 2019-09-11

**Authors:** Sonia Menon, Joel Francis, Natasha Zdraveska, Alfred Dusabimana, Samit Bhattacharyya

**Affiliations:** 1 Faculty of Medical and Health Sciences, Ghent University, De Pintelaan 185 P3, 9000 Ghent, Belgium; 2 Noncommunicable Diseases Unit, Department of Public Health, Institute of Tropical Medicine Antwerp, Antwerp, Belgium; 3 Department of Family Medicine and Primary Care, University of the Witwatersrand, Johannesburg, South Africa; 4 Department of Epidemiology and Biostatistics, Muhimbili University of Health and Allied Sciences, Dar es Salaam, Tanzania; 5 Faculty of Pharmacy, Department of Clinical Pharmacy, Saints Cyril and Methodius University, Skopje, Republic of Macedonia; 6 Epidemiology and Social Medicine, University of Hasselt, Hasselt, Belgium; 7 Department of Mathematics, School of Natural Sciences, Shiv Nadar University, Greater Noida, India

**Keywords:** Tuberculosis, Diabetes mellitus, Intransient hyperglycaemia, Protocol

## Abstract

**Background:**

Diabetes mellitus (DM) is burgeoning as a global chronic health condition. Some studies suggest that tuberculosis (TB) can even cause diabetes in those not previously known to be diabetic, which as a corollary can add to the already heavy global DM burden. The World Health Organization (WHO) recommends screening for DM at the start of TB treatment; however, it remains to be elucidated which patients with TB-induced hyperglycaemia are at risk for developing DM and who would benefit from a more regular follow-up. This systematic review will aim to firstly synthesise literature on the irreversibility of TB-induced hyperglycaemia in individuals with previously undiagnosed type 2 diabetes mellitus and secondly to synthesise literature on risk factors for progression from TB-induced hyperglycaemia to overt DM in previously undiagnosed.

**Methods:**

We will search for relevant studies in electronic databases such as PubMed, EMBASE, PROQUEST, and SCOPUS. Furthermore, references will be hand searched to identify other studies. A flow diagram will be drawn to identify the studies retrieved from each database. We will review all publications that include studies containing data on impaired glucose metabolism upon TB diagnosis, and the quality of all eligible studies will be assessed using the Newcastle-Ottawa Scale. We will further conduct a meta-analysis to pool estimates on the risk of progression of persistent hyperglycaemia to overt DM within this population group, as well as the risk factors for this progression. We will use a random effect model to assess heterogeneity, will carry out sensitivity analysis to explore the influence of a single study on the overall estimate, and will report our findings from our systematic review and meta-analysis according to PRISMA guidelines. Egger’s test will be performed to explore the presence of selective reporting bias. If data allow, we will perform a subgroup/meta-regression analysis. Summary effects will be reported using odds ratio, hazard ratio, and relative risk ratios. Furthermore, any clinical, epidemiological, and public health research gaps we identify will be described in a research proposal.

## Background

In 2016, there were an estimated 10.4 million new tuberculosis (TB) cases across the globe, with India and followed by Indonesia, China, Philippines, Pakistan, Nigeria, and South Africa accounting for 64% of the total burden [1, 2]. Whilst global efforts aimed to combat tuberculosis (TB) have saved an estimated 53 million lives since 2000 and reduced the TB mortality rate by 37%, according to the Global TB Report 2018, TB remains the infectious disease which caused the highest number of deaths in 2017 [2, 3].

Concurrently, diabetes mellitus (DM) is burgeoning as a global chronic health condition, which can be attributed to increases in obesity and changing patterns of diet and physical activity as well as ageing [3]. The global prevalence of diabetes among adults over 18 years of age has risen from 4.7% in 1980 to 8.5% in 2014, and by 2030, its prevalence is expected to increase by 50% [4].

As is the case with TB, three in four diabetic patients live in low-income countries [5, 6], and according to the World Health Organization (WHO), in 2015, there were an estimated 1.6 million deaths that could be attributed to diabetes [4]. This is all the more worrisome as DM is in its own right a risk factor for cardiovascular disease (CVD), an umbrella term for a set of clinical conditions that affect 13 million people worldwide, accounting for 25% of all deaths [7]. Previous studies have shown that enhanced DM case management and improved blood glucose control can lead to improved TB treatment outcomes [8, 9]. As an added benefit, this helps to alleviate the economic burden within the healthcare systems on a global level.

Whilst a meta-analysis has shown that DM increases the risk of TB threefold compared to the non-diabetic population [10], and a pooled overall prevalence of tuberculosis in diabetic patients was calculated to be 4.72% (95% CI 3.62–5.83%) [11], there is still a lack of knowledge whether TB can predispose an individual to DM. Infections, including TB, often worsen glycaemic control in diabetic patients [12]. Several cross-sectional studies have reported an association between TB outcome and the presence of hyperglycaemia [13, 14]. The transitory hyperglycaemia is likely to be of multifactorial aetiology and may be explained by the occurrence of early phase hyperglycaemia, induced by rifampicin administration [15, 16], hyperglycaemia that normally accompanies corticosteroid administration [17], or stress hyperglycaemia ensuing from an intricate interplay between disturbed cytokine and hormone production, resulting in excessive hepatic glucose production and insulin resistance [18].

Some studies suggest that TB can even cause diabetes in those not previously known to be diabetic [19–21]. In persons with prolonged glucose impairment, this may set in motion a vicious cycle, in which poorly controlled diabetes might in turn augment the severity of infections [22] and the risk of MDR TB and cardiovascular disease.

Due to the worsening of DM and TB in low- and middle-income countries, the WHO and the International Union Against Tuberculosis and Lung Disease (IUATLD) support a collaborative framework that recommends bidirectional screening including testing for TB among people with DM [23]. The WHO recommends screening for DM at the start of TB treatment; however, little is known which patients are at risk for developing DM and who would benefit from a more regular follow-up.

We aim to build on the literature review by Magee et al. [24] on the epidemiology of intransient hyperglycaemia in TB patients with previously undiagnosed DM 2 individuals by performing a systematic review to synthesise the literature on the epidemiology of stress-induced hyperglycaemia with no date limit. Secondly, we will synthesise literature on risk factor for the above progression. We will then quantify these relationships using a meta-analysis. Both our narrative review and meta-analysis will allow us to identify clinical, epidemiological, and public health research gaps. Our protocol has been registered with PROSPERO (CRD42019118173; https://www.crd.york.ac.uk/PROSPERO/display_record.php?RecordID=118173).

## Methods

### Literature search strategy

Our search strategy, selection of publications, and reporting of results for the review will be conducted in accordance with the PRISMA guidelines [25]. The domains of the search terms are Tuberculosis AND hyperglycaemia OR Diabetes Mellitus. We will systematically search relevant studies in the following electronic databases: PubMed, EMBASE, PROQUEST, and SCOPUS without any language restrictions. Reference lists of all retrieved articles will be checked to identify further eligible publications.

### Eligibility criteria

For eligibility, only peer-reviewed publications will be included. Eligible studies should report on TB-induced hyperglycaemia in all individuals irrespective of their age and have outcomes measured prospectively more than once. Retrospective case control studies where DM or pre-DM are cases will be included. In addition, the systematic review will report on risk factors for persistent TB-induced hyperglycaemia in newly detected DM. Case studies and cross-sectional studies will not be eligible.

### Types of participants

Participants will be individuals newly diagnosed with DM following a previous TB diagnosis.

### Outcome measures

TB disease is defined by a positive chest x-ray and a sample of sputum [26].

Overt, clinically manifested diabetes is preceded by prediabetes, a metabolic state characterised by elevated blood glucose levels in the range between normal glycaemia and diabetes, which is indicative of a risk for progression to type 2 diabetes.

Prediabetes is defined as impaired fasting glycemia (IFG; fasting plasma glucose (FPG) levels ≥ 100 mg/dl (5.6 mmol/l)) or impaired glucose tolerance (IGT; 2-h oral glucose tolerance test (OGTT) of ≥ 140 mg/dl (7.8 mmol/l)) [27]. Independent risk factors for irreversible TB-induced hyperglycaemia in patients screened for DM following TB diagnosis will be reported.

### Data extraction and management

Two investigators (SM and JMF) will independently review and critically evaluate all identified studies (citations) for inclusion. SM and JMF will extract the data from eligible studies independently using Excel spreadsheet. SM and JMF will resolve the discrepancies on eligible studies and extracted data through a discussion involving SB and AD. The following items will be recorded: first author, study period, publication year, study type, study population, total sample size, age, method of diagnosis of TB, time of follow-up, glucose impairment status when diagnosed with TB, method used to ascertain persistent hyperglycaemia, risk factors for persistent DM, and confounders adjusted for. Whenever provided, outcome measures will be extracted along with its associated 95% confidence interval. In case of any missing information or need for clarification, respective study authors will be contacted via email.

### Risk of bias in individual studies

Our systematic review will be conducted strictly in accordance with a protocol. We will assess the quality of the studies using the Newcastle-Ottawa Scale [28]. The risk of bias will be ascertained by two reviewers, using the Cochrane Risk of Bias Tool [29]. According to the Cochrane Risk of Bias Tool, each selected study will be graded as low, high, or unclear risk of bias across different types of bias. Assessment will be performed at the study level and will focus on selection, performance, detection, attrition, and reporting biases. The risk of bias for each included study will be taken into consideration during data synthesis. A sensitivity analyses, excluding the studies that will be graded as high or unclear risk of bias to determine the effect of removal on the results, will be performed.

### Data analysis

We will assess all identified articles for quality and perform quantitative synthesis of the information obtained from the eligible articles. In a situation whereby only a few (less than three) articles qualify for quantitative synthesis, we will then perform narrative synthesis.

As TB-induced hyperglycaemia is not rare, we will not be able to consider hazard ratios, risk ratios, and odds ratios as the same. If the data permit, we will classify studies according to their type of effect size and consequently pool these different effect size estimates. To pool effect sizes, we will be using STATA 15 and present the descriptive findings using tables and the meta-analysis findings using forest plots. Symmetry of funnel plots and Egger’s test will be performed to explore the presence of selective reporting bias.

We will use random effect model to assess heterogeneity, as it is a conservative approach and data is expected to vary across studies. We will assess heterogeneity using the Desmordian-Laid test *I*^2^ (*I*^2^ ≥ 75%) which equates to high heterogeneity and visual inspection of the forest plot for inconsistencies in effect sizes and their confidence intervals. Meta-regression will be performed if data permit to analyse for sources of heterogeneity. In addition to a synthesis of literature, if heterogeneity permits, we will quantify this relationship using a meta-analysis.

## Discussion

To the best of our knowledge, this systematic review and meta-analysis will provide the first narrative synthesis and quantitative estimate of the risk of irreversible TB-induced hyperglycaemia in those newly screened for DM upon TB diagnosis.

The pooled estimate will guide clinicians and policymakers in informing patients and governments about the risk of persistent hyperglycaemia in hitherto undiagnosed DM patients with TB and will likely aid the identification of patients at risk who will be required for more regular follow-up. Moreover, it will provide an estimate of the future global DM burden. Importantly, this systematic review will enable the identification of clinical, epidemiological, and public health gaps, thus outlining directions for further investigation.

The findings from the review will be disseminated in a peer-reviewed journal, and we will recommend or carry out research to bridge the identified gaps.

### Strengths and limitations

A major strength of our study is the comprehensive review within four major databases in order to include all potential articles. Anticipated limitations include the heterogeneity in the sample size of the retrieved studies and quality of the study design; therefore, it is difficult to give the right weight to the current evidence. Furthermore, heterogeneity in the sensitivity or specificity of screening methods may also render results less comparable. Also, there may be a lack of prospective studies with a clear strategy to deal with the loss to follow-up retrieved by the literature search.

Significant and positive research is more likely to be published, and the negative or insignificant findings will be less likely included in our systematic review due to the fact that they are not publicly available for inclusion. Additionally, our review will include studies published in English language. Consequently, several relevant unpublished or published studies in a language other than English may be been excluded.

## Data Availability

N/A

## Abbreviations

DM: Diabetes mellitus
FPG: Fasting plasma glucose
HIV: Human immunodeficiency virus
IGT: Impaired glucose tolerance
IUATLD: International Union Against Tuberculosis and Lung Disease
MDR TB: Multidrug-resistant TB
TB: Tuberculosis
WHO: World Health Organization

## References

[1] World Health Organisation. WHO report signals urgent need for greater political commitment to end tuberculosis 2018 [Available from: https://www.who.int/news-room/detail/30-10-2017-who-report-signals-urgent-need-for-greater-political-commitment-to-end-tuberculosis.

[2] Global Tuberculosis Report 2018. Geneva: World Health Organisation; 2018.

[3] Hu FB, Manson JE, Stampfer MJ, Colditz G, Liu S, Solomon CG (2001). Diet, lifestyle, and the risk of type 2 diabetes mellitus in women. N Engl J Med..

[4] Word Health Organisation. Tuberculosis and diabetes 2016 [Available from: https://www.who.int/tb/publications/diabetes_tb.pdf.

[5] International Diabetes Foundation. IDF Diabetes Atlas 8th Edition 2017 [Available from: https://www.idf.org/e-library/epidemiology-research/diabetes-atlas/134-idf-diabetes-atlas-8th-edition.html.

[6] Wild S, Roglic G, Green A, Sicree R, King H (2004). Global prevalence of diabetes: estimates for the year 2000 and projections for 2030. Diabetes Care..

[7] Lozano R, Naghavi M, Foreman K, Lim S, Shibuya K, Aboyans V (2012). Global and regional mortality from 235 causes of death for 20 age groups in 1990 and 2010: a systematic analysis for the Global Burden of Disease Study 2010. Lancet.

[8] Baker MA, Harries AD, Jeon CY, Hart JE, Kapur A, Lonnroth K (2011). The impact of diabetes on tuberculosis treatment outcomes: a systematic review. BMC Med..

[9] Lo HY, Yang SL, Lin HH, Bai KJ, Lee JJ, Lee TI (2016). Does enhanced diabetes management reduce the risk and improve the outcome of tuberculosis?. Int J Tuberc Lung Dis..

[10] Jeon CY, Murray MB (2008). Diabetes mellitus increases the risk of active tuberculosis: a systematic review of 13 observational studies. PLoS Med..

[11] Wagnew F, Eshetie S, Alebel A, Dessie G, Tesema C, Abajobir AA (2018). Meta-analysis of the prevalence of tuberculosis in diabetic patients and its association with cigarette smoking in African and Asian countries. BMC Res Notes..

[12] Dooley KE, Chaisson RE (2009). Tuberculosis and diabetes mellitus: convergence of two epidemics. Lancet Infect Dis..

[13] Bailey SL, Ayles H, Beyers N, Godfrey-Faussett P, Muyoyeta M, du Toit E (2016). The association of hyperglycaemia with prevalent tuberculosis: a population-based cross-sectional study. BMC Infect Dis..

[14] Kibirige D, Ssekitoleko R, Mutebi E, Worodria W (2013). Overt diabetes mellitus among newly diagnosed Ugandan tuberculosis patients: a cross sectional study. BMC Infect Dis..

[15] Sharma TN, Agarwal KC, Gupta PR, Purohit SD, Sharma VK, Mathur BB (1986). Further experience on glucose tolerance test during rifampicin therapy. J Assoc Physicians India..

[16] Takasu N, Yamada T, Miura H, Sakamoto S, Korenaga M, Nakajima K (1982). Rifampicin-induced early phase hyperglycemia in humans. Am Rev Respir Dis..

[17] Moreira J, Castro R, Lamas C, Ribeiro S, Grinsztejn B, Veloso VG (2018). Hyperglycemia during tuberculosis treatment increases morbidity and mortality in a contemporary cohort of HIV-infected patients in Rio de Janeiro, Brazil. Int J Infect Dis..

[18] Dungan KM, Braithwaite SS, Preiser JC (2009). Stress hyperglycaemia. Lancet..

[19] Abrass Christine K. (1991). Fc receptor-mediated phagocytosis: Abnormalities associated with diabetes mellitus. Clinical Immunology and Immunopathology.

[20] Nichols GP. Diabetes among young tuberculous patients; a review of the association of the two diseases. Am Rev Tuberc. 1957;76:1016–30. 10.1164/artpd.1957.76.6.1016 13488012

[21] Zack MB, Fulkerson LL, Stein E. Glucose intolerance in pulmonary tuberculosis. Am Rev Respir Dis. 1973;108:1164–69. 10.1164/arrd.1973.108.5.1164 4746573

[22] PRK L, Henry MK, Melmed S, Polonsky KS (2003). Williams’ textbook of endocrinology.

[23] The International Union Against Tuberculosis and Lung Disease and World Health Organization collaborative framework for care and control of tuberculosis and diabetes. Geneva: World Health Organization; 2011. 26290931

[24] Magee MJ, Salindri AD, Kyaw NTT, Auld SC, Haw JS, Umpierrez GE (2018). Stress hyperglycemia in patients with tuberculosis disease: epidemiology and clinical implications. Curr Diab Rep..

[25] Moher D, Liberati A, Tetzlaff J, Altman DG, Group P (2009). Preferred reporting items for systematic reviews and meta-analyses: the PRISMA statement. PLoS Med..

[26] Centre of Disease Control. Tuberculosis Testing & Diagnosis Atlanta: Centre of Disease Control; 2016 [Available from: https://www.cdc.gov/tb/topic/testing/default.htm

[27] Wong E, Backholer K, Harding J, Gearon E, Stevenson C, Freak-Poli R (2012). A systematic review and meta-analysis of diabetes and risk of physical disability and functional impairment - protocol. Syst Rev..

[28] Wells GAS, B.; O'Connell, D.; Peterson, J.; Welch, V.; Losos, M.; Tugwell,P. The Newcastle-Ottawa Scale (NOS) for assessing the quality of nonrandomised studies in meta-analyses 2018 [Available from: http://www.ohri.ca/programs/clinical_epidemiology/oxford.asp.

[29] Higgins JP, Altman DG, Gotzsche PC, Juni P, Moher D, Oxman AD (2011). The Cochrane Collaboration’s tool for assessing risk of bias in randomised trials. BMJ..

